# Effects of reasoning demands triggered by genre on Chinese EFL learners' writing performance

**DOI:** 10.3389/fpsyg.2023.1164262

**Published:** 2023-05-17

**Authors:** Cheng Peng, Zhen Bao

**Affiliations:** School of Foreign Languages, Shanghai Jiao Tong University, Shanghai, China

**Keywords:** task complexity, L2 writing performance, the cognition hypothesis, the trade-off hypothesis, expository writing, argumentative writing

## Abstract

**Introduction:**

Genres, having distinct communicative functions, elicit different levels of reasoning demands in writing tasks. The current study investigated the influence of cognitive complexity triggered by a seldom studied pair of genres (expository writing vs. argumentative writing) on Chinese advanced EFL learners' writing performance.

**Method:**

A total of 76 L2 learners participated in two writing tasks: one simpler expository writing task involving fewer reasoning demands and the other more complex argumentative writing task eliciting more reasoning demands. Multiple measure indices were adopted to comprehensively reflect the differences in production dimensions between the two writing tasks, such as lexical complexity, syntactic complexity, accuracy, fluency, and cohesion.

**Results and discussion:**

The results showed that cognitive complexity significantly improved lexical complexity, clausal complexity, and cohesion, which generally supported the Cognition Hypothesis. However, phrasal structures and clausal structures within the construct of syntactic complexity displayed a trade-off effect, partially corroborating the Trade-off Hypothesis. Accuracy and fluency were uninfluenced, verifying neither of these hypotheses. Implications for sequencing and designing L2 writing tasks were provided for relevant stakeholders.

## 1. Introduction

As second language acquisition theories and task sequencing criteria develop (Xu et al., 2022), the effect of task complexity on L2 writing performance has been examined by many studies, generating conflicting results (e.g., Ong and Zhang, 2010; Rahimi and Zhang, 2018; Zhan et al., 2021). Further research is warranted to deepen our understanding of the conceptualization and operationalization of writing task complexity and to help instructors and assessors appropriately design and sequence writing tasks based on L2 learners' proficiency (Robinson, 2015). Furthermore, it remains to be examined whether L2 writers' attentional resources are sufficient when completing writing tasks of different cognitive complexity.

Revolving around the question, Robinson (2001) proposed the cognition hypothesis that an increase in task complexity could improve L2 production quality. Motivated by the hypothesis, this study was conducted to investigate L2 writing performance across a simple task (expository writing involving a lower level of reasoning) and a complex task (argumentative writing involving a higher level of reasoning). The task complexity was manipulated via reasoning demands elicited by genres, whose effects on lexical complexity, syntactic complexity, accuracy, fluency, and cohesion were investigated.

Two hypotheses concerning task complexity proposed by Skehan and Foster (1997, 1999, 2001) and Robinson (2001, 2015) served as the theoretical foundations for this study. Similar to Kellogg's (1996) L1 writing model, Skehan and Foster (1997) proposed the trade-off hypothesis that attentional resources are limited during the L2 production process. However, Robinson (2001) proposed a contrasting hypothesis, i.e., the cognition hypothesis. In keeping with previous L2 writing studies (e.g., Rahimi, 2019), this study set out to verify the effects of task complexity, manipulated via writing genre, on L2 writing performance, based on the aforementioned two hypotheses.

## 2. Review of the literature

### 2.1. Theoretical background

Writing tasks play an important role in writing improvement and language development. As assumed in the output hypothesis (Swain, 1985), writing tasks could promote L2 learning by making learners realize the “gap” between what they want to write and what they can write, and this “gap” will motivate learners to learn more target language to modify their written output. Furthermore, during the writing process, L2 learners are forced to consider not only the semantic but also the syntactic aspects to generate legitimate and comprehensible language.

Writing tasks could activate and orchestrate various cognitive resources during three sub-processes: formulation, execution, and monitoring, according to Kellogg's (1996) model. Among the three processes, the formulation was theorized to place the most demands on the working memory, followed by monitoring. Formulation entails planning content to be written and translating it into words. While planning, writers deploy attentional resources to generate and organize content coherently. During translation, lexical and syntactic forms are accessed and encoded to express the content into words. Although Kellogg's model was initially proposed for L1 writing tasks, it was also confirmed to be applicable to L2 writing tasks (e.g., Kormos, 2011; Révész et al., 2017).

In task-based language learning, learners are required to allocate their attentional resources to meet tasks' cognitive demands. The two most influential theories concerning the influence of cognitive demands on L2 production are the trade-off hypothesis and the cognition hypothesis. These two theories make contrary predictions about the relationship between task cognitive demands and language production. Although the two hypotheses were originally put forward for L2 oral production, previous studies have confirmed their applicability to L2 writing (e.g., Rahimi and Zhang, 2018; Zhan et al., 2021).

The trade-off hypothesis was put forward by Skehan and Foster (1997, 1999, 2001). They hypothesized that attentional resources and processing capacity are limited, so learners have to prioritize one aspect of language production over others, hence triggering trade-off effects among complexity, accuracy, and fluency. For example, when an increase in cognitive task complexity triggers over-taxation of attentional resources, learners will give priority to meaning and content planning over linguistic forms, leading to increased fluency but decreased complexity and accuracy.

Contrarily, Robinson (2001, 2005) proposed that task complexity was associated with the cognitive demands imposed on learners, but learners could pay attention to multiple facets of language production simultaneously by drawing on multiple attentional resource pools, thus promoting interlanguage learning and development. Task complexity could be manipulated along resource-directing and resource-dispersing dimensions. Increasing task complexity along the resource-directing dimension (by placing cognitive demands on learners) could lead to improved accuracy, improved complexity, and decreased fluency, whereas increasing task complexity along the resource-dispersing dimension (by placing performative demands on learners) could result in lower accuracy, lower complexity, and lower fluency.

Task complexity is assumed to lie in the formulation process, in which content planning and linguistic encoding place great cognitive demands on learners (Robinson, 2005). In the present study, the argumentative writing task requires more reasoning demands, because learners have to conceptualize the content by reasoning, analyzing the controversial issue, and arguing for or against one side with supportive evidence. By contrast, an expository writing task just involves learners presenting information about one campus activity based on their prior knowledge, so the content can be accessed from learners' long-term memory with ease.

### 2.2. Reasoning demands triggered by genre and L2 writing performance

So far, due to the inconsistent operationalizations of task complexity and the use of different language complexity indices, research findings regarding the effects of reasoning demands triggered by genres on L2 writing performance have been conflicting.

A line of research partially supported the trade-off hypothesis. For example, Way et al. (2000) investigated the effects of different genres (i.e., descriptive, narrative, and expository) on L2 learners' writing performance. The results indicated that the syntactic complexity (e.g., T-unit length) was higher in expository essays than that in descriptive or narrative ones. However, an almost reverse trend (descriptive > narrative > expository) was detected in fluency and accuracy measures. Thus, as the reasoning demands increased, the trade-off effect existed among syntactic complexity, accuracy, and fluency, which supported the trade-off hypothesis.

Another line of research partially supported the cognition hypothesis. To further examine the influence of genre, Lu (2011) employed 14 syntactic complexity measures. He found that argumentative essays generally displayed more complex syntactic features than narrative ones. Yoon and Polio (2017) replicated Lu's (2011) study and included other dimensions such as lexical complexity, accuracy, and fluency. Their results revealed that the syntactic complexity in argumentative writing was generally higher than that in narrative writing, as evidenced by the length of production and phrasal complexity. However, no significant effect was detected in clausal complexity, accuracy, and fluency. Based on the above two studies (Lu, 2011; Yoon and Polio, 2017), Zhan et al. (2021) found similar results. Specifically, the argumentative writing exhibited higher syntactic complexity (length of production and phrasal structures) and fluency than did the narrative writing, but there were no significant differences in lexical complexity or accuracy.

More comprehensively, Yang (2014) examined the effect of task complexity on L2 writing performance across four writing genres (narrative, expository, expo-argumentative, and argumentative). Yang found that accuracy, fluency, lexical diversity, and lexical sophistication were not significantly influenced; the lexical density of expository writing was the highest, while that of narrative writing was the lowest. The syntactic complexity can be ranked as argumentative > expository > narrative (the indices of expo-argumentative writing fluctuated). The general writing performance can be summarized as non-narrative > narrative.

All the above-mentioned four studies (i.e., Lu, 2011; Yang, 2014; Yoon and Polio, 2017; Zhan et al., 2021) revealed that when more reasoning demands were imposed, the produced syntactic complexity, in particular, would increase, partially corroborating the cognition hypothesis.

There still existed some other studies supporting neither of these two hypotheses. Contrary to previous studies' findings, Ruiz-Funes (2013) investigated the effects of task complexity on 24 intermediate L2 learners' writing performance, detecting non-significant differences for all measures across two writing tasks (narrative vs. expository). Again, Ruiz-Funes (2014) examined the influence of task complexity on eight advanced L2 learners' writing production, still finding no significant differences for all indices across two writing tasks (expository vs. argumentative). Although the pair of expository and argumentative writing tasks was studied, the sample size was very small, decreasing its statistical power and generalizability.

In light of different operationalizations of task complexity, it is hardly possible to compare different research results simply based on the broad categories of “less complex writing task” and “more complex writing task.” Therefore, we narrow down the concept of task complexity to cognitive complexity brought about by genre in writing tasks. Nevertheless, the findings concerning writing performance influenced by different genres are still not consistent across studies, probably due to such confounding factors as the topic, learner proficiency, and the use of different sets of measure indices. It is, therefore, necessary to control these confounding factors to only focus on the influence of genres and their embedded reasoning demands.

On the other hand, though previous studies did examine genre effects in writing tasks (e.g., narrative vs. non-narrative; argumentative vs. non-argumentative), few studies are setting out to explicitly compare expository writing and argumentative writing among L2 advanced learners. In China, these two major writing genres, whose knowledge has been imparted and constantly applied during secondary and tertiary education, play a pivotal role in writing pedagogy, learning, and assessment. In academic practice, college students frequently need to formally explain concepts/information to others (corresponding to the purpose of expository writing) or to argue for/against someone's viewpoint with supporting evidence to be convincing (corresponding to the purpose of argumentative writing). Yet, the lack of studies explicitly centering on these two writing genres fails to provide pedagogical or assessment implications for these two vital types of writings, though these implications are very practical and essential.

If informed of the differences in writing performance caused by these two genres' distinct reasoning demands, relevant stakeholders will be benefited. For example, L2 instructors will be more expert at arranging or sequencing writing tasks by taking task complexity induced by reasoning demands into consideration, to promote the development of EFL learners' writing ability. Similarly, L2 assessors could be better at anchoring the validity and reliability of writing assessments involving these two genres. Furthermore, EFL learners will more consciously deploy specific linguistic features characteristic of each genre in their writings, to better fulfill the genre-related communicative functions.

To shed light on the role of the reasoning demands elicited by these two genres in writing tasks, this study set out to investigate the influence of task complexity induced by these two genres (i.e., expository writing vs. argumentative writing) on L2 writing performance. Task complexity in our study was manipulated as previous studies did (e.g., Yang, 2014; Zhan et al., 2021), and our study focused on two seldom-examined writing tasks. In addition, participants in our study were advanced Chinese EFL learners, who were seldom investigated in previous studies. Moreover, multidimensional measures of lexical complexity and syntactic complexity were adopted, which is of great importance (Norris and Ortega, 2009; Johnson, 2017). In addition, cohesion indices were also included in this study to assess the macro-level organization of written production and learners' higher-order writing skills so as to address the concern expressed by Kuiken and Vedder (2008) that the improved linguistic dimensions of L2 writing might compete with other higher-order dimensions of writing. Finally, our study classified syntactic complexity into phrasal complexity and clausal complexity, which were less explicitly examined.

Accordingly, the present study answered the following research question: How does the cognitive complexity triggered by genre influence advanced EFL learners' writing performance in terms of lexical complexity, syntactic complexity, accuracy, fluency, and cohesion?

## 3. Method

### 3.1. Participants

A total of 76 undergraduate sophomores majoring in the English language were recruited from a top university in Shanghai, China, using convenience sampling. These students got writing feedback and were awarded bonus credits based on their writing performance. Eleven participants were removed as they failed to follow the researchers' instructions. The essays of 65 students were retained for further analysis and research. Among them, there are 43 female and 22 male students, whose ages ranged from 19 to 21 years (M = 19.86, SD = 9 months). All of the participants' mother tongues were Mandarin Chinese.

The participants are engaged in a language learning program, which includes courses in linguistics, English literature, English culture, and language skills. They have been learning English as a foreign language in the classroom setting for over 13 years, but none of them have any overseas study experiences. The Oxford Quick Placement Test (version 2.0) was used to assess students' English proficiency. As a result, all of the students' placement scores fell into the advanced proficiency range (46–57 out of 60), M = 50.95, SD = 2.56.

### 3.2. Writing tasks

Different writing genres will place different cognitive demands on EFL learners (Yoon and Polio, 2017). Expository writing and argumentative writing are two discourse types with distinct communicative purposes. Thus, an expository writing task and an argumentative writing task adapted from the Chinese CET-Band 4 and Chinese TEM-Band 4, respectively, were employed in this study (see writing prompts in Supplementary material). College English Tests (CET) designed for non-English major college students and Test for English Majors (TEM) for English major college students are both standardized English proficiency tests in China, whose reliability and validity have been examined and well-documented (Yan and Huizhong, 2006; Yan and Jinsong, 2011). It is universally accepted in China that TEM-Band 4 is much more difficult than CET-Band 4.

The participants completed the tasks in two English classes (one task each day) under the supervision of their English teacher. Students were asked to work on their own and were not allowed to use cellphones, dictionaries, or reference textbooks. Since the writing tasks were regarded as completely independent of each other, no consideration was taken concerning the practice effect of one task over the other. In this study, the time allotted for each task was 30 min.

Cognitive task complexity is associated with reasoning demands induced by genres (Ruiz-Funes, 2015). The tasks that involve more reasoning demands were thought to be more cognitively complex in EFL writing studies (Rahimi, 2019). In this study, the tasks were designed to elicit different levels of cognitive complexity. Both tasks were created around the theme of activities. In Task 1, students were instructed to introduce an impressive college activity based on their prior knowledge in an expository manner. Task 2 required students to argue for or against the phenomenon (volunteer activities) mentioned in the prompt by giving supporting evidence. In terms of the reasoning demands, Task 2 was considered to impose higher cognitive loads than Task 1.

To further validate the task complexity, five experienced EFL writing instructors and five doctoral postgraduates majoring in linguistics were invited to judge the complexity of these two writing tasks on a Likert scale, which ranged from 1 (extremely simple) to 9 (extremely complex). The expert ratings were in line with the categorization of task complexity in this study.

### 3.3. Dependent variables

The dependent variables consist of lexical complexity, syntactic complexity, accuracy, fluency, and cohesion. Task production quality is typically measured by the indices of complexity, accuracy, and fluency (e.g., Foster and Skehan, 1996; Norris and Ortega, 2009). However, there is no consensus on the measures of writing quality to date (Johnson, 2017). Researchers have adopted different indices to assess learners' writing production. The necessity of using multiple indices of linguistic complexity to investigate L2 writing quality was pointed out by Norris and Ortega (2009). Thus, to avoid the inconsistency of evaluating indices, this study adopted a set of comprehensive indices to evaluate different dimensions of writing production.

#### 3.3.1. Lexical complexity

Lexical complexity, a multidimensional construct, can be categorized into three subcategories: lexical diversity, lexical sophistication, and lexical density, but very few studies examined the latter two subcategories (Johnson, 2017). This study examined all three subcategories.

Since the index type/token ratio (TTR) is sensitive to sample size and length (Rahimi, 2019), corrected type/token ratio (CTTR) was employed to measure lexical diversity, countering the influence of sample size and length effect (Ong and Zhang, 2010; Zhan et al., 2021). To comprehensively assess lexical diversity, two other well-validated indices, *D*-value and measure of textual lexical diversity (MTLD), were also adopted, which were employed in previous studies (e.g., Révész et al., 2017; Rahimi, 2019).

In addition, previous studies have found that psycholinguistic values, such as the age of acquisition (AoA) and concreteness, are important indicators of lexical sophistication (De Wilde et al., 2020). The log frequency for content words was shown to be more reliable to indicate lexical sophistication than the raw frequency (Just and Carpenter, 1980). This study adopted these three indices to examine lexical sophistication, which can predict the quality of L2 writing (Zhang et al., 2022). Finally, lexical density (LD) was employed to gain a big picture of learners' lexical complexity.

#### 3.3.2. Syntactic complexity

Syntactic complexity, also a multidimensional construct, could be analyzed from different syntactic facets. In line with the previous classification of syntactic complexity (Bulté and Housen, 2014; Kyle and Crossley, 2018), apart from measuring production length, the present study measured syntactic complexity at other two levels: phrasal complexity and clausal complexity.

In this study, multiple indices were adopted to capture the genres' effects on syntactic complexity by following previous studies (e.g., Lu, 2011; Rahimi and Zhang, 2019; Zhan et al., 2021). The first syntactic index (i.e., STS) measures the extent of structural similarity in the text using Coh-Metrix 3.0 (McNamara et al., 2010), which reflects the overall syntactic sophistication. The higher the index is, the less diverse the syntactic structures are. The other 11 syntactic indices obtained from the Syntactic Complexity Analyzer (Lu, 2011) focus on three dimensions: three indices concerning the length of the unit, e.g., mean length of sentence (MLS), mean length of T-unit (MLT), and mean length of clause (MLC); three indices measuring clausal complexity, e.g., clauses per T-unit (C/T), dependent clauses per clause (DC/C), and dependent clauses per T-unit (DC/T); five indices calculating phrasal complexity, e.g., verb phrases per T-unit (VP/T), complex nominals per T-unit (CN/T), complex nominals per clause (CN/C), coordinate phrases per T-unit (CP/T), and coordinate phrases per clause (CP/C).

#### 3.3.3. Accuracy

Accuracy refers to lexical and grammatical correctness in learners' essays, measured by errors made in learners' writing, but the errors are not concerned with punctuation, spelling, or capitalization, which are not typical issues among advanced EFL learners. In this study, the number of errors per T-unit and the number of errors per 100 words were adopted to measure the learner's accuracy, both of which were employed and verified in previous studies (e.g., Ruiz-Funes, 2015). The higher the ratios are, the less accurate learners' essays are.

#### 3.3.4. Fluency

Since the writing time was controlled for all learners, the total number of words was used as one index of fluency (Johnson et al., 2012). In addition, fluency was also assessed by the other index, i.e., the mean number of words per T-unit, which was regarded as a reliable fluency index by Wolfe-Quintero et al. (1998). The two indices combined can better capture the fluency of learners' writing production.

#### 3.3.5. Cohesion

Cohesion proved to be one of the important indicators of L2 writing performance (Crossley et al., 2016; Zhang et al., 2022). Cohesive devices play a vital role in connecting ideas in writing (Halliday and Hasan, 1976). Previous research has validated and confirmed the efficacy of Coh-Metrix indices (i.e., latent semantic analysis, co-reference, and connectives) in evaluating textual cohesion (Foltz et al., 1998; McNamara et al., 2010).

Latent semantic analysis (LSA) is a statistical representation of textual cohesion by evaluating the level of semantic similarity between sentences and paragraphs (Foltz, 1996). In this study, both local and global LSA indices were used to measure the conceptual similarity between sentences and paragraphs, respectively.

Co-reference was measured by stem overlap and content word overlap, both of which are more inclusive compared with noun overlap or argument overlap (Crossley et al., 2016). Stem overlap refers to how often a noun in one sentence shares a common lemma with another content word in another sentence. Content word overlap calculates the number of shared content words between sentences.

Connectives are vital signal words of relations in essays and thus promote discourse cohesion. The appropriate use of connectives can improve textual organization and content unity (Halliday and Hasan, 1976). To reflect the overall use of connectives in learners' essays, we adopted the holistic index to identify all connectives used in learners' writings.

### 3.4. Statistical analyses

First, 11 students' essays were excluded due to not following requirements, so the two writing tasks of 65 students were analyzed. Then, the 130 essays were typed in Microsoft Word documents and coded by researchers via the Lexical Complexity Analyzer (Lu, 2012), Syntactic Complexity Analyzer (Lu, 2011), and Coh-Metrix 3.0 (McNamara et al., 2010) to obtain lexical complexity, syntactical complexity, fluency, and cohesion indices. Paired samples *t*-tests were carried out to check the differences between the two writing tasks' performance indices, with the alpha level set at 0.05 for all tests. Cohen's *d* was adopted to measure the effect size, and the standards were followed: *d* = 0.2–0.4, a small effect size; *d* = 0.5–0.7, a medium effect size; and *d* >0.8, a large effect size (Cohen, 1988). The program SPSS 21.0 was employed for the abovementioned statistical analyses.

## 4. Results

### 4.1. Effects on learners' lexical complexity

The paired samples *t*-tests detected a series of task effects on lexical complexity (see Table 1). First, the mean differences between the two lexical diversity indices were both statistically significant. For example, as for *D*-value, MD = −12.96, *p* = 0.001, *d* = 0.81; and as for MTLD, MD = −10.85, *p* = 0.04, *d* = 0.47. It meant that EFL learners were more likely to use more diverse lexical forms when completing the complex writing task (i.e., argumentative writing). Furthermore, the complex writing task elicited significantly more abstract words than did the less complex task, MD = 50.30, *p* = 0.000, *d* = 2.14. Task effect was also reflected in AoA, MD = −76.12, *p* = 0.000, *d* = 2.72, which indicated that learners tended to use later acquired words in the more complex task. Moreover, the effect sizes for the above *t*-tests were generally large. These lexical indices indicated that participants tended to deploy diverse and sophisticated vocabulary when dealing with more complex writing tasks. As task complexity increased, the lexical complexity generally showed an upward trend.

**Table 1:** Comparison of lexical complexity in Task 1 (exposition) and Task 2 (argumentation).

**Sub-categories**	**Indices**	**Task 1**		**Task 2**		**MD**	***p***	***d***
		**M**	**SD**	**M**	**SD**			
Lexical diversity	CTTR	6.59	1.05	6.21	0.53	0.39	0.09	0.39
	*D*-value	79.50	15.76	92.46	16.76	−12.96	0.001	0.81
	MTLD	75.44	19.28	86.29	17.18	−10.85	0.04	0.47
Lexical sophistication	Log freq	3.02	0.08	3.00	0.09	0.02	0.41	0.18
	AoA	310.64	17.78	386.76	25.04	−76.12	0.000	2.72
	Concreteness	415.43	19.17	365.13	12.10	50.30	0.000	2.14
Lexical density	LD	0.52	0.03	0.52	0.03	0.00	0.76	0.07

CTTR, corrected type/token ratio; D-value, lexical variability based on Malvern and Richards (1997); MTLD, measure of textual lexical density; Log freq, log frequency for content words; AoA, age of acquisition for content words; Concreteness, concreteness for content words; LD, lexical density.

In addition, the paired samples *t*-tests failed to reveal the effects of task complexity on other indices of lexical complexity. As for CTTR, log frequency, and LD, none of the mean differences between the two tasks were statistically significant.

### 4.2. Effects on learners' syntactic complexity

As indicated in Table 2, regarding the structural similarity, argumentative writing presented significantly fewer similar syntactic structures than did expository writing, MD = 0.02, *p* = 0.004, *d* = 0.70, which showed that advanced learners were more likely to vary their syntactic structures in more complex writing task. Regarding the length of production, only MLC in Task 1 was significantly higher than that in Task 2, and the effect size was very large (MD = 2.07, *p* = 0.000, *d* = 1.06), which meant that learners produced longer clauses in the simple writing task than in the complex one.

**Table 2:** Comparison of syntactic complexity in Task 1 (exposition) and Task 2 (argumentation).

**Sub-categories**	**Indices**	**Task 1**		**Task 2**		**MD**	***p***	***d***
		**M**	**SD**	**M**	**SD**			
Overall sophistication	STS	0.10	0.03	0.08	0.02	0.02	0.004	0.70
Mean length of unit	MLS	19.21	5.23	20.34	6.45	−1.13	0.51	0.15
	MLT	17.51	4.95	18.30	6.43	−0.79	0.64	0.10
	MLC	11.88	2.69	9.81	1.44	2.07	0.000	1.06
Clausal complexity	DC/T	0.48	0.24	0.81	0.39	−0.33	0.005	0.70
	DC/C	0.31	0.11	0.42	0.08	−0.11	0.001	0.87
	C/T	1.48	0.28	1.86	0.56	−0.38	0.01	0.58
Phrasal complexity	VP/T	1.88	0.36	2.77	0.76	−0.89	0.000	0.98
	CN/T	2.30	0.76	1.90	0.61	0.40	0.050	0.46
	CN/C	1.57	0.46	1.03	0.21	0.54	0.000	1.41
	CP/T	0.52	0.30	0.39	0.17	0.13	0.01	0.61
	CP/C	0.36	0.23	0.22	0.10	0.14	0.002	0.77

STS, structural similarity; MLS, mean length of sentence; MLT, mean length of T-unit; MLC, mean length of the clause; DC/T, dependent clauses per T-unit; DC/C, dependent clauses per clause; C/T, clauses per T-unit; VP/T, verb phrases per T-unit; CN/T, complex nominals per T-unit; CN/C, complex nominals per T-unit; CP/T, coordinate phrases per T-unit; CP/C, coordinate phrases per clause.

Additionally, increasing task complexity had a significant influence on EFL learners' clausal complexity features. The mean values of three indices in Task 2 were all significantly higher than those in Task 1, with large effect sizes (C/T, MD = −0.38, *p* = 0.01, *d* = 0.58; DC/C, MD = −0.11, *p* = 0.001, *d* = 0.87; DC/T, MD = −0.33, *p* = 0.005, *d* = 0.70), which meant that the more complex writing task elicited more clausal constructions.

**Figure 1:**
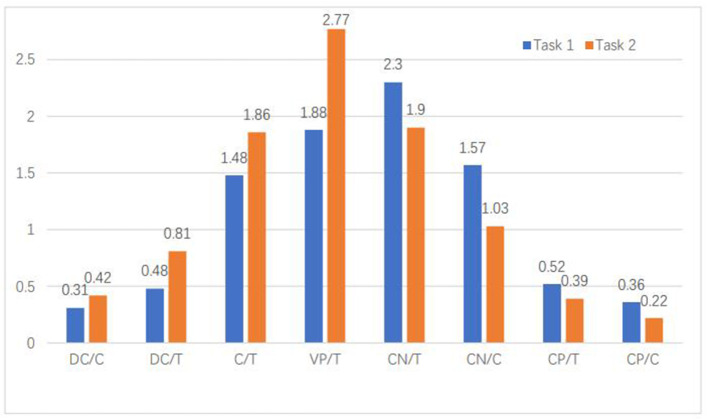
Clausal and phrasal complexity indices' comparison.

Contrarily, advanced EFL learners tended to generate fewer phrasal structures (except VP/T) in complex writing tasks. Instead, they tended to employ more phrasal devices (especially nominals) to convey and present information in the simple writing task (i.e., expository writing). All phrasal indices showed significant differences between the two writing tasks with large effect sizes. It can be shown from Figure 1 that there existed a trade-off effect between phrasal complexity and clausal complexity.

In summary, with the increase of reasoning demands in EFL learners' writing tasks, the number of phrasal structures significantly decreased (especially nominals), whereas the number of clausal structures significantly increased.

### 4.3. Effects on learners' writing accuracy and fluency

Task complexity had no significant effect on learners' writing accuracy or fluency, as shown in Table 3. Neither index of accuracy displayed statistically significant differences between the two writing tasks, e.g., MD = −0.10, *p* = 0.43, *d* = 0.17 (Etot/T); MD = 0.02, *p* = 0.97, *d* = 0.00 (NER). Similarly, neither fluency index yielded evidence of significant differences, e.g., MD = −0.80, *p* = 0.64, *d* = 0.10 (W/T); MD = 1.14, *p* = 0.98, *d* = 0.01 (TNW).

**Table 3:** Comparison of accuracy and fluency in Task 1 and Task.

	**Task 1**		**Task 2**		**MD**	***p***	***d***
	**M**	**SD**	**M**	**SD**			
Etot/T	0.68	0.25	0.78	0.56	−0.10	0.43	0.17
NER	4.23	2.26	4.21	1.86	0.02	0.97	0.00
W/T	17.50	4.94	18.30	6.43	−0.80	0.64	0.10
TNW	386.52	177.36	385.38	60.77	1.14	0.98	0.01

Etot/T, number of errors per T-unit; NER, number of errors per 100 words; W/T, words per T-unit; TNW, total number of words.

### 4.4. Effects on learners' writing cohesion

With the increase in task complexity, the essays generated in the complex task were more cohesive than those in the simple task, as evidenced by both implicit and explicit measures (see Table 4). The implicit measures, i.e., LSA-p, SO-s, and CWO-s, all showed significant differences between the two writing tasks with medium effect sizes. The explicit measure, i.e., ACI, revealed that the difference in the use of connectives between the two writing tasks was statistically significant with a large effect size (MD = −20.41, *p* = 0.000, *d* = 1.15). In short, advanced EFL learners tended to employ more cohesive devices in the more reason-demanding writing task (i.e., argumentative writing).

**Table 4:** Comparison of cohesion in Task 1 and Task 2.

	**Task 1**		**Task 2**		**MD**	***p***	***d***
	**M**	**SD**	**M**	**SD**			
LSA-s	0.18	0.07	0.16	0.05	0.01	0.42	0.18
LSA-p	0.31	0.11	0.41	0.10	−0.09	0.005	0.69
SO-s	0.33	0.14	0.40	0.19	−0.07	0.05	0.44
CWO-s	0.07	0.03	0.09	0.02	−0.03	0.000	0.78
ACI	80.09	16.73	100.50	15.32	−20.41	0.000	1.15

LSA-s, Latent Semantic Analysis overlap (at sentence level); LSA-p, Latent Semantic Analysis overlap (at paragraph level); SO-s, Stem overlap (at sentence level); CWO-s, Content word overlap (at sentence level); ACI, All connectives incidence.

## 5. Discussion

### 5.1. Effects on lexical complexity

Increasing cognitive complexity had significantly positive influences on lexical diversity (*D*-value and MTLD) and lexical sophistication (AoA and concreteness). With the increase in reasoning demands, advanced EFL learners' lexical complexity in writing also increased, which supported Robinson's cognition hypothesis.

Advanced EFL learners tended to employ more diverse and sophisticated words when dealing with the cognitively demanding task, probably because simple words could not meet the demands of a complex writing task, which involved a deeper level of form-conceptualization mapping. In addition, advanced learners had a good knowledge of L2 vocabulary, and the complex task provided them with a channel of lexical production. Advanced learners may be more skilled at funneling their attentional resources toward the lexical forms while conceptualizing and organizing content to be written (Rahimi, 2019). Learners' lexical density remained constant across two writing tasks, which may indicate that learners' high proficiency pushed their content words' proportion to the limit, reaching the ceiling effect.

On the one hand, some of the results regarding lexical complexity corroborated previous studies. For example, the findings concerning CTTR supported Kuiken and Vedder's (2008) and Zhan et al.'s (2021) studies, which found no significant difference either. Regarding frequency, the findings were consistent with Kormos's (2011) and Johnson et al.'s (2012) studies in which task complexity did not have significant effects on lexical frequency.

On the other hand, our results also refuted previous findings. Concerning *D*-value and concreteness, our results contradicted the study of Kormos (2011), who conducted two narrative writing tasks and found that *D*-value and concreteness decreased significantly with the increase of cognitive complexity. Regarding *D*-value and MTLD, our findings were not in line with Révész et al. (2017), who manipulated task complexity via content support. The inconsistent results may be caused by different manipulations or operationalizations of task complexity.

Our findings regarding general lexical complexity were inconsistent with some previous studies (e.g., Kuiken and Vedder, 2008; Zhan et al., 2021), which may be due in part to the use of different lexical indices. Specifically, relatively few studies adopted sophisticated lexical indices, such as *D*-value and MTLD, which were considered more sensitive to the change of task complexity and less influenced by text length compared with TTR and its transformations (Johnson, 2017). Also, very few studies included multiple lexical complexity indices to reflect the effects of task complexity on all three dimensions of lexical complexity, i.e., lexical diversity, sophistication, and density. Many studies only examined the lexical diversity indices, which might not be sensitive enough to capture the task complexity's effects on lexical complexity, thus resulting in inconsistent findings.

To sum up, the set of comprehensive indices in this study indicated that lexical complexity would increase with the increase of reasoning demands.

### 5.2. Effects on syntactic complexity

The structural similarity index, reflecting the overall syntactic complexity, showed that the syntactic structures in the complex writing task were more varied, partially due to the use of more clauses.

The results also indicated that the clauses in the complex writing task (i.e., argumentative writing) were significantly shorter (as shown by MLC) because more clauses/dependent clauses were embedded in essays produced in the complex task, as revealed by significantly higher DC/T, C/T, and DC/C in argumentative writing. As for the simple writing task, the significantly longer clauses were attributable to the fact that more phrases were embedded in clauses, as reflected by statistically higher CP/C and CN/C in expository writing. EFL learners employed more phrases, instead of clauses or dependent clauses (correspondingly, fewer verb phrases), to pack and condense more information in expository writing (see Table 5).

**Table 5:** Summary of syntactic complexity across two writing tasks.

**Task type**	**Genre type**	**Clause length**	**Clause density**	**Phrase density**	**Phrasal constituents**
Task 1	Expository	Longer	Lower	Higher	More nominal phrases; fewer verb phrases
Task 2	Argumentative	Shorter	Higher	Lower	Fewer nominal phrases; more verb phrase

Phrasal complexity and clausal complexity did not increase simultaneously but competed with each other. As phrasal complexity increased in expository writing, the clausal complexity would fall. On the contrary, when phrasal complexity decreased in argumentative writing, the clausal complexity would rise. Our findings also corroborated the findings in Yang's (2014) and Lei et al.'s (2023) studies, whose clausal complexity exhibited an increasing trend from expository writing to argumentative writing. Contrarily, the nominal phrasal complexity presented a downward trend with the increase in reasoning demands.

Previous researchers also found a trade-off effect between phrasal and clausal complexities in other pairs of writing tasks. For example, Biber et al. (2011) found that informative writing tended to show more phrasal structures and fewer clausal structures, while spoken discourse displayed a reverse trend. The simple writing task in our study also required an informational presentation of certain school activities, thus presenting more phrasal features; by contrast, similar to the communicative function characteristic of conversation, the complex writing task in our study had a persuasive purpose, thus showing more clausal features.

A similar trade-off effect could also be found across narrative and argumentative writing tasks in previous studies (Lu, 2011; Yoon and Polio, 2017; Zhan et al., 2021). With the increase of reasoning demands triggered by genre, the phrasal complexity indices increased, while the clausal complexity indices decreased, probably because learners needed to employ different kinds of syntactic structures to meet distinct reasoning demands induced by genre-related communicative functions.

The findings concerning syntactic complexity can be interpreted in both weak and strong manners. The weak interpretation is that both genres of essays were complex in syntax but in different dimensions. In expository writing, learners were more likely to generate phrasal structures to pack more information into relatively longer clauses. In argumentative writing, which required a higher level of reasoning about others' motivations for doing something controversial, learners preferred to use cognitive state terms accompanied by clausal structures, e.g., *somebody thought that, somebody wondered whether*. No obvious increase of holistic syntactic complexity can be inferred from the weak interpretation, thus not supporting the cognition hypothesis.

The strong explanation for the findings is based on the developmental progression hypothesis (Biber et al., 2011, 2016). In L2 development, clausal structures are acquired at relatively earlier stages and represent a lower level of syntactic complexity. By contrast, complex phrasal embedding is produced in later stages toward adulthood, which is considered to represent a higher level of syntactic complexity. Thus, it can be cautiously concluded that the syntactic complexity decreased (with fewer phrasal features and more clausal features) as task complexity increased. Therefore, the findings were aligned with the trade-off hypothesis.

As noted by Ellis and Yuan (2004), when learners were composing essays, they gave priority to the access of lexical items over the generation of syntactic structures, which meant that the cognitive resources allotted to syntactic complexity were limited. The cognitive loads imposed on limited working memory in a timed condition could easily result in a trade-off effect. It can be inferred that advanced learners in our study would resort to clausal devices to relieve cognitive loads when reasoning demands increased, to funnel more attentional resources to higher-order writing skills, e.g., content conceptualization and textual organization. On the contrary, when a task imposed fewer reasoning demands, learners could allocate more available cognitive resources to retrieve or construct phrasal structures, which is conceived as more challenging.

To sum up, due to the limited attentional resources, participants could only attend to some dimension of syntactic complexity, especially when the reasoning demands involved in tasks increased. The trade-off effect between clausal complexity and phrasal complexity supported the trade-off hypothesis.

### 5.3. Effects on accuracy and fluency

On the one hand, the two accuracy indices (Etot/T and NER) in our study proved to be uninfluenced by task complexity, supporting previous studies (Kormos, 2011; Ruiz-Funes, 2015; Yoon and Polio, 2017; Zhan et al., 2021). However, our results contradicted the results of Rahimi and Zhang's (2018) and Rahimi's (2019) studies, both of which showed that accuracy significantly decreased when task complexity increased. In addition, our results also refuted Kuiken and Vedder's (2007) and Yang's (2014) studies, which found that accuracy significantly increased when task complexity increased.

On the other hand, the findings for fluency indicated that task complexity did not influence fluency, confirming the findings of Révész et al.'s (2017) and Yoon and Polio's (2017) studies. However, the results were not consistent with some previous research findings (e.g., Yang, 2014; Rahimi and Zhang, 2018; Zhan et al., 2021) that the increase in task complexity had significantly positive impacts on fluency measures. The inconsistent findings regarding accuracy and fluency could be attributed to the adoption of different measure indices and different manipulations of task complexity.

This result did not support the trade-off hypothesis or the cognition hypothesis, which may be caused by learners' insensitivity to respond to different reasoning demands across these two writing tasks or the possibility that the cognitive demands of the two tasks, in the view of the learners, were not different enough to generate distinct performances in accuracy and fluency. Furthermore, that could be because the influences of reasoning demands on accuracy and fluency are insignificant in magnitude compared with that of learners' L2 proficiency, which was well-controlled for in this study (i.e., the participants were homogenous in proficiency). As revealed by Way et al. (2000), L2 proficiency level significantly influenced writing accuracy and fluency across different writing genres. Learners of the same L2 proficiency were expected to consistently produce similar language quality with similar fluency across different writing tasks (Norris and Ortega, 2009; Guo et al., 2013).

### 5.4. Effects on cohesion

The findings in this study indicated that task complexity induced by different reasoning demands had a significant impact on cohesion. In our study, the complex writing essays (i.e., argumentative essays) were found to be more coherent than the simple ones (i.e., expository essays). Specifically, the former featured a higher level of LSA (global), lexical overlap, and connective devices. The results supported Rahimi's (2019) findings that cohesion in essays would improve with the increase of task complexity manipulated via reasoning demands. However, our findings were not consistent with Kormos's (2011) and Révész et al.'s (2017) studies, which found that there were no significant effects of task complexity (manipulated via content support) on cohesion across two writing tasks. The difference in the operationalization of task complexity may lead to inconsistent findings.

The findings concerning cohesion partially supported the cognition hypothesis. As task complexity increased, the cohesive features increased along with lexical complexity and clausal complexity. Although the cognition hypothesis did not explicitly predict the influence of task complexity on textual cohesion, simultaneous improvements in cohesion and linguistic complexity indicated that increasing task complexity triggered by reasoning demands could enhance L2 writing quality, confirming the spirit of the cognition hypothesis.

The reasoning demands in complex tasks prompted L2 learners to utilize multiple resource pools to process different dimensions of L2 production simultaneously. The complex writing task could motivate L2 learners to produce more complex linguistic forms (i.e., micro-level lexical and clausal complexity) and meanwhile to pay attention to higher-order writing skills (i.e., macro-level organizational features), consequently enhancing the overall writing quality. The findings addressed the concern expressed by Kuiken and Vedder's (2008).

## 6. Conclusion and limitations

Considering the paucity of task complexity research into the comparison between expository writing and argumentative writing, our study aimed to investigate the effects of manipulating task complexity (±reasoning demands) on Chinese advanced EFL learners' writing production. The findings showed that increasing task complexity, as manipulated via reasoning demands elicited by genre, generally improved L2 writing performance. Specifically, the essays in the complex writing task (i.e., argumentative writing) exhibited increased lexical complexity and clausal complexity, as well as more cohesive features. However, the increase in task complexity also led to the use of fewer phrasal structures in the complex writing task. Additionally, the accuracy and fluency were not influenced by the increase in task complexity.

Theoretically, these findings overall supported the cognition hypothesis in that increasing reasoning demands led to improvements in lexical complexity, clausal complexity, and textual cohesion. However, within the construct of syntactic complexity, there existed a trade-off effect between phrasal structures and clausal structures, which also supported the trade-off hypothesis. In terms of the constancy of accuracy and fluency measures, neither the cognition hypothesis nor the trade-off hypothesis was corroborated, probably because the two hypotheses are aimed at the influence of task complexity on speaking performance, rather than writing performance.

Methodologically, different coding tools were utilized to measure the same construct to improve measuring reliability, such as the Lexical Complexity Analyzer, Syntactic Complexity Analyzer, and Coh-Metrix. In addition, multi-dimensional/fine-grained operationalizations of one linguistic construct were employed, e.g., seven indices were used to measure lexical complexity. Moreover, the study explicitly classified syntactic complexity into phrasal complexity and clausal complexity, which was frequently proposed in previous studies (Staples et al., 2016; Yoon and Polio, 2017; Kyle and Crossley, 2018) but was not widely adopted.

Pedagogically, EFL instructors and assessors should consider cognitive demands when assigning writing tasks and designing writing assessments. EFL writing tasks should be sequenced from the simple to the complex in terms of the involved reasoning demands, so as to promote L2 learning and interlanguage development (Robinson, 2015). Tasks requiring fewer reasoning demands should be completed before those requiring more demands. Compared with the process of simply introducing an activity based on prior knowledge, arguing for or against an issue with justifiable evidence consumed more cognitive resources. Since advanced EFL learners were able to generate more complex language in the complex writing task, they should be given more chances to perform complex writing tasks, so as to promote L2 development through output. Moreover, considering that producing phrasal structures and clausal structures simultaneously might overwhelm learners' limited attentional resources, teachers might as well develop and adopt instructional strategies to train learners to use advanced syntactic structures packed with more information (e.g., clausal structures embedded with phrases), which would be helpful for learners to retrieve these advanced structures.

There are some limitations in this study. First, since subjects in this study fell into the advanced L2 proficiency range, the findings cannot be generalized to learners belonging to other proficiency levels. Second, as for fluency measures, although writing time was held constant for all learners, there was a possibility that some learners wrote more quickly and finished ahead of the time limit. Therefore, the findings regarding the effect of task complexity on fluency should be consulted cautiously. Third, we conducted a series of paired-samples t-tests for multiple comparisons without applying the Bonferroni adjustment, which might increase the probability of Type I errors. Considering the concern, we rechecked the statistical analyses using Bonferroni adjusted alphas (e.g., alpha for lexical complexity set at 0.05/7 = 0.0071; alpha for syntactic complexity set at 0.05/12 = 0.0042; alpha for cohesion set at 0.05/5 = 0.01), and found that some indices' test results would become non-significant with alphas above the thresholds (e.g., MTLD, DC/T, CN/T, CP/T). Nevertheless, these indices could still reflect the systematic changes between the two writing tasks' productions. Besides, the overall writing performance discrepancies can be captured through the lens of other alternative indices which measured the same construct in nature (e.g., D-value, DC/C, CN/C, CP/C). In short, we arrived at the same conclusion using either original alphas or adjusted alphas. Future researchers may consider conducting a general linear model (MANOVA) to control for multiple within-participant comparisons. Finally, the trade-off effect between clausal complexity and phrasal complexity calls for more future research to examine it across writing tasks of different cognitive complexity.

## Data availability statement

The raw data supporting the conclusions of this article will be made available by the authors, without undue reservation.

## Ethics statement

Ethical review and approval was not required for the study on human participants in accordance with the local legislation and institutional requirements. Written informed consent for participation was not required for this study in accordance with the national legislation and the institutional requirements.

## Author contributions

CP was responsible for conducting the research, writing, and revising the manuscript. ZB was responsible for analyzing the data and revising the manuscript. CP and ZB responded to all the reviewers' comments and edited the final version of the manuscript.
