# Supplementary material for: Effects of reasoning demands triggered by genre on Chinese EFL learners' writing performance
Source: Front Psychol. 2023 May 17;14:1164262. doi: 10.3389/fpsyg.2023.1164262 (PMC10231673; doi:10.3389/fpsyg.2023.1164262)
Supplement: Supplementary file 1 [file Presentation_1.pdf]

Supplementary Material (Appendix)

Effects of Reasoning Demands Triggered by Genre on Chinese EFL Learners' Writing Performance

Cheng Peng, Zhen Bao*

*** Correspondence:** Corresponding Author: baozhen2020@sjtu.edu.cn

1 Appendix

Simple writing task prompt (adapted from CET-Band 4 in 2014)

Directions: For this part, you are allowed 30 minutes to write an expository essay about an activity that has impressed you most in college. In our college, there are various kinds of wonderful activities, and our students benefit a lot from them. You need to choose one activity you have participated in and give a detailed introduction of it. In you essay, you'd better include the impressive features of the activity, the role you played in it and the significance it brought to you.

Please write at least 200 words, and marks will be awarded for content relevance, content sufficiency, organization, language quality. Failure to follow the above instructions may result in a loss of marks.

Complex writing task prompt (adapted from TEM-Band 4 in 2010)

Directions: For this part, you are allowed 30 minutes to write an argumentative essay about your attitude towards a phenomenon on college campus. It was recently reported that some college students actively participated in a lot of volunteer activities to help others and enhance their own social skills, only to overlook their own studies, which has led to heated debates as to whether college students should participate in volunteer activities. You need to analyze the issue and different opinions, and then give your own view and reasons for your view.

Please write at least 200 words, and marks will be awarded for content relevance, content sufficiency, organization, language quality. Failure to follow the above instructions may result in a loss of marks.
